# Unveiling Hidden Variations in Lung Fissures: A Cadaveric Perspective on Surgical and Diagnostic Challenges

**DOI:** 10.7759/cureus.108078

**Published:** 2026-04-30

**Authors:** Bhoopesh Raja, Manoj M Kulkarni, Hetal Vaishnani, Aditya A Musale, Jahnavi Nath

**Affiliations:** 1 Anatomy, Smt. B. K. Shah Medical Institute and Research Centre, Sumandeep Vidyapeeth Deemed to Be University, Vadodara, IND

**Keywords:** cadaver, craig and walker classification, lobar anatomy, lung fissures, thoracic surgery

## Abstract

Introduction

Anatomical variations in lung fissures arise from incomplete obliteration of intersegmental planes during embryogenesis, resulting in incomplete, accessory, or absent fissures. These variants pose challenges in thoracic surgery, increasing risks of air leaks, prolonged operative times, and conversion to thoracotomy, while complicating radiological interpretation.

Materials and methods

This descriptive cross-sectional study aimed to characterise the patterns and completeness of major lung fissures in adult cadaveric lungs and to compare these features between the right and left sides. Sixty unpaired adult cadaveric lungs (30 right, 30 left) were examined in the Department of Anatomy, Smt. B. K. Shah Medical Institute and Research Centre, Sumandeep Vidyapeeth Deemed to Be University, Vadodara, Gujarat, India. Each lung was examined for fissure number, completeness, accessory fissures, and lobar demarcation. Fissure completeness was classified using the Craig and Walker grading system (Grade I-IV), with findings recorded and photographically documented.

Results

Of 30 right lungs, 19 (63.3%) showed normal fissure patterns while 11 (36.7%) exhibited abnormalities. Of the 30 left lungs, 25 (83.3%) were normal, and five (16.7%) showed abnormal morphology. The difference in normal fissure prevalence between sides was not statistically significant (p = 0.143, Fisher’s exact test). Complete oblique fissures were present in all right lungs but in 83.3% of left lungs (p = 0.052).

Conclusion

Cadaveric lungs demonstrate notable fissural variation, with abnormalities more frequent in the right lungs. Recognition of graded fissure completeness is essential for surgeons and radiologists to optimise preoperative planning and minimise complications.

## Introduction

The human respiratory system is fundamentally dependent on the functionality of the lungs, which are principal organs located within the thoracic cavity. These paired organs are indispensable for respiration, moving freely within the thoracic cage except at the hilum. The thoracic cavity is enclosed by the sternum and ribs, providing essential protection. The approximate weight of the right and left lungs is 625 g and 565 g, respectively. Anatomically, the lung is a conical organ with an apex, base, two surfaces, and three borders. The left lung is smaller than the right; the position of the diaphragm determines lung length, while the position of the heart influences lung width [1-4].

Embryologically, lung fissure development begins around the sixth week of gestation, when bronchopulmonary segments are delineated by intersegmental spaces. These spaces normally obliterate except along certain planes, resulting in oblique and horizontal fissures. Failure of complete obliteration leads to incomplete or accessory fissures [5,6]. Such anatomical deviations can result in atypical lobar patterns, often incidental findings in dissection or radiological imaging.

From a surgical perspective, knowledge of fissure anatomy is critical in planning segmentectomy or lobectomy procedures. Incomplete fissures may lead to air leaks, haemorrhage, or complications in identifying bronchovascular structures during thoracic surgeries. Moreover, radiological misinterpretation of accessory fissures can mimic pathological lines or lesions, leading to diagnostic pitfalls [7-9]. Fissure completeness is commonly graded using the system proposed by Craig and Walker, which categorises fissures from Grade I (complete) to Grade IV (fused) [10]. Incomplete fissures increase surgical complications: 23.3% persistent air leaks vs. 0% in complete fissures, longer operative times (248 vs. 174 min), chest drainage (11.8 vs. 6.3 days), hospital stays (12.6 vs. 7.1 days), and higher thoracotomy conversion (13.3% vs. 6%) [10,11]. These findings highlight the clinical importance of preoperative identification of fissure completeness. Hence, a detailed understanding of both normal and variant fissure patterns is essential for anatomists, radiologists, and thoracic surgeons alike.

Therefore, the present study aimed to systematically characterise the patterns and completeness of major lung fissures in adult cadaveric lungs using the Craig and Walker classification, and to compare the prevalence of normal and abnormal fissure configurations between right and left lungs.

## Materials and methods

This descriptive cross-sectional cadaveric study was conducted in the Department of Anatomy at Smt. B. K. Shah Medical Institute and Research Centre, Sumandeep Vidyapeeth Deemed to Be University, Vadodara, Gujarat, India. The study was approved by the university ethics committee (SVIEC/ON/medi/SRP/Aug/25/1).

Sixty unpaired adult human lungs (30 right, 30 left) were obtained from routine dissection laboratory cadavers. Specimens were collected consecutively from routine undergraduate dissection sessions between August 2023 and July 2024. Right and left lungs from the same cadaver were included whenever both were available and met the inclusion criteria; in some cases, only one lung from a cadaver fulfilled the criteria. Identification of right versus left lung was confirmed at the time of retrieval by hilar anatomy (arrangement of the main bronchus, pulmonary artery, and pulmonary veins) and by the number of lobes, rather than by the number or pattern of fissures alone; any specimen in which side identification could not be confirmed was excluded. Sex and age information were not consistently documented and were therefore not analysed. Inclusion criteria included adult lungs (older than 18 years), adequate preservation, and absence of gross trauma or pathology that distorted the fissures. Specimens with tumours, consolidation, adhesions, or gross surgical or traumatic disruption were excluded a priori and did not contribute to the final sample of 60 lungs. All lungs were fixed in 10% formalin.

Each lung underwent external inspection to ascertain the following: presence of oblique fissures (both right and left lungs), presence of horizontal fissures (right lung only), completeness of each fissure along its expected course, and presence of any apparent accessory fissures. All lungs were examined on a stainless-steel dissection table under standard laboratory lighting. Fissure presence and completeness were assessed visually and by gentle blunt probing along the expected fissure lines. Observations were performed independently by two anatomists (one faculty member and one postgraduate trainee), and any discrepancies in grading were resolved by consensus during the same session; formal inter‑observer reliability statistics were not calculated. Any accessory fissure (for example, azygos, superior or inferior accessory, or lingular fissures) and any aberrant horizontal-type fissure identified on the left lung were planned to be recorded and described separately and interpreted outside the Craig-Walker grading system.

Fissures were subsequently categorised as follows: Complete - extending from the hilum to the lung border without any parenchymal fusion; Incomplete - fissure visible only in part of its length, with parenchymal fusion for the remaining segment; Absent/fused - no visible fissure line externally.

For descriptive purposes, a right lung was operationally considered to have a “normal” fissure pattern when both a complete oblique (Craig-Walker Grade I) and a complete horizontal fissure (Grade I) were present; a left lung was considered “normal” when a single complete oblique fissure (Grade I) was present. Any deviation from these Grade I configurations on either side was classified as “abnormal.” We recognise that, under the Craig-Walker system, Grade II (complete visceral cleft with parenchymal fusion only at the base) represents near-complete rather than genuinely incomplete anatomy; however, as no Grade II specimens were encountered in the present series, this distinction did not affect the practical classification in this study.

Craig and Walker grading

The completeness of each major fissure was graded using Craig and Walker’s classification system: Grade I - complete fissure with lobes completely separated; Grade II - complete visceral cleft with parenchymal fusion at the base; Grade III - incomplete visceral cleft; Grade IV - complete fusion without any apparent fissure. In the present study, specimens in which no external fissure line was identifiable, and the parenchyma appeared uninterrupted along the expected plane, were operationally classified as Grade IV, consistent with “complete fusion without any apparent fissure” as described in the original system.

High-resolution photographs were captured from multiple perspectives for representative specimens, including one normal right and one normal left lung as reference. Photographs were obtained using a standard digital camera under uniform laboratory lighting and were edited only for global adjustments of brightness and contrast, without any alteration of anatomical details. Cadaveric lungs display inherent variation in three-dimensional shape, surface contour, pleural texture, and apparent fullness, reflecting natural inter-individual anatomical differences as well as the effects of formalin fixation; consequently, even when oriented apex-up, individual specimens may appear visually different across figures, although their fissure morphology has been classified consistently using the Craig-Walker system. Each specimen in this series was assigned a unique identifier (Specimen #1-60); the photographed specimens carry their identifier within the figure, and the corresponding side, pattern category, Craig-Walker grades, and specimen-specific morphological features for all 60 lungs are tabulated in the Appendices. Data were manually entered into master charts for the right and left lungs. Descriptive statistics (frequencies and percentages) were calculated for fissure patterns and Craig-Walker grades. The proportion of normal vs. abnormal fissure patterns was compared between right and left lungs using Fisher’s exact test. Fissure completeness (Grade I vs. non‑Grade I) was similarly compared between sides for oblique fissures and within right lungs for horizontal fissures. Although the present study is primarily descriptive, Fisher’s exact test was applied to quantify side-to-side differences in fissure completeness, given the small expected cell counts; statistical inferences from these comparisons are reported with caution in light of the modest sample size and resulting limited statistical power. All statistical analyses were performed using IBM SPSS Statistics for Windows, Version 26 (Released 2018; IBM Corp., Armonk, New York, United States), and a p-value < 0.05 was considered statistically significant.

## Results

A total of 30 right lungs and 30 left lungs were examined to determine fissure completeness and categorise observed patterns. Results are presented separately for right and left lungs, followed by comparative analysis.

**Figure 1:**
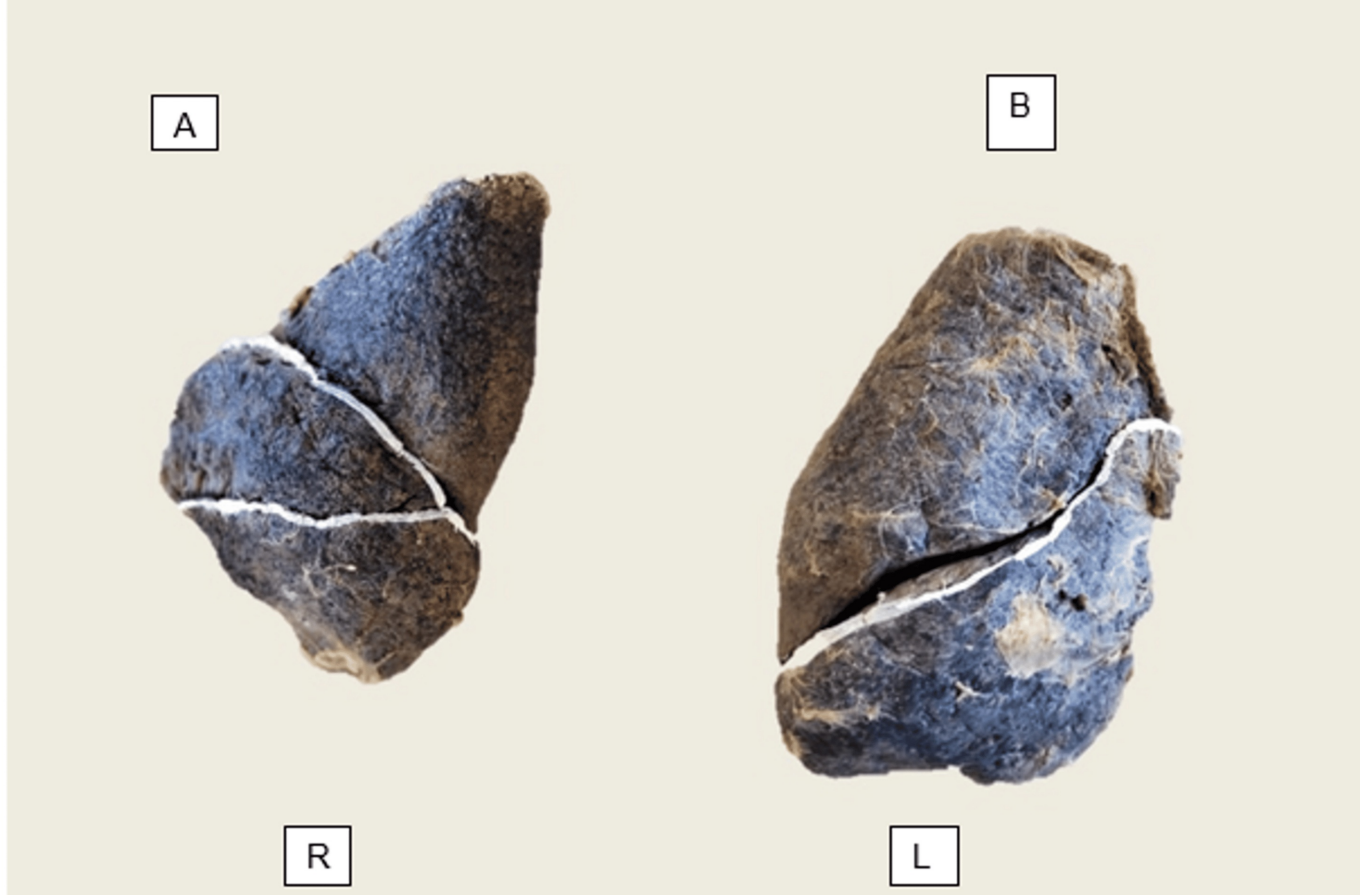
Normal lung fissure anatomy. (A) Right lung (labelled R) Specimen #5 showing complete oblique fissure separating upper and lower lobes, and complete horizontal fissure separating upper and middle lobes (Craig-Walker Grade I for both). (B) Left lung (labelled L) Specimen #35 showing a single complete oblique fissure separating upper and lower lobes (Craig-Walker Grade I). Both specimens are shown in anatomical orientation (apex up, diaphragmatic surface down).

A normal lung pattern can be seen in Figure 1.

Patterns of major fissures in the right lungs

**Figure 2:**
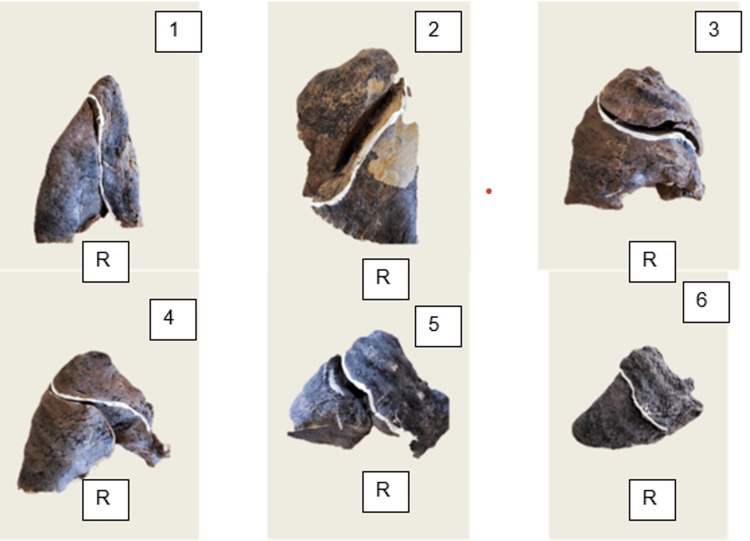
Right lung (R) specimens with bilobed configuration (n = 6, Specimens #4, #11, #17, #22, #27, #30 - corresponding to sub-panels 1-6, respectively). Each panel is numbered (1-6) and oriented apex-up. A single complete oblique fissure separates the upper and lower lobes; the horizontal fissure is absent (Craig-Walker Grade I oblique, Grade IV horizontal).

Among the 30 right lung specimens analysed, 19 (63.3%) displayed the classical pattern characterised by complete oblique and horizontal fissures. Six lungs (20.0%) presented only a single complete oblique fissure while completely lacking the horizontal fissure, resulting in a bilobed configuration (Figure 2).

**Figure 3:**
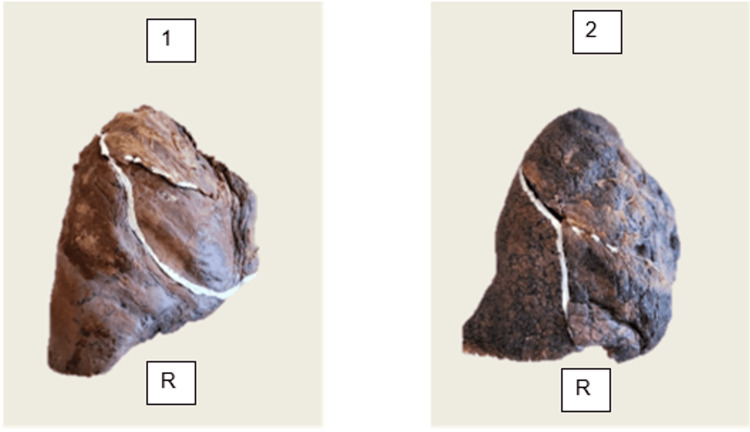
Right lung (R) with complete oblique fissure and completely fused horizontal fissure (n = 2; Specimens #9 and #20, corresponding to sub-panels 1 and 2, respectively). The horizontal fissure is represented only by a shallow surface groove (Craig-Walker Grade I oblique, Grade IV horizontal).

Furthermore, two lungs (6.7%) exhibited a complete oblique fissure accompanied by a completely fused horizontal fissure, with only a shallow surface groove present (Figure 3).

**Figure 4:**
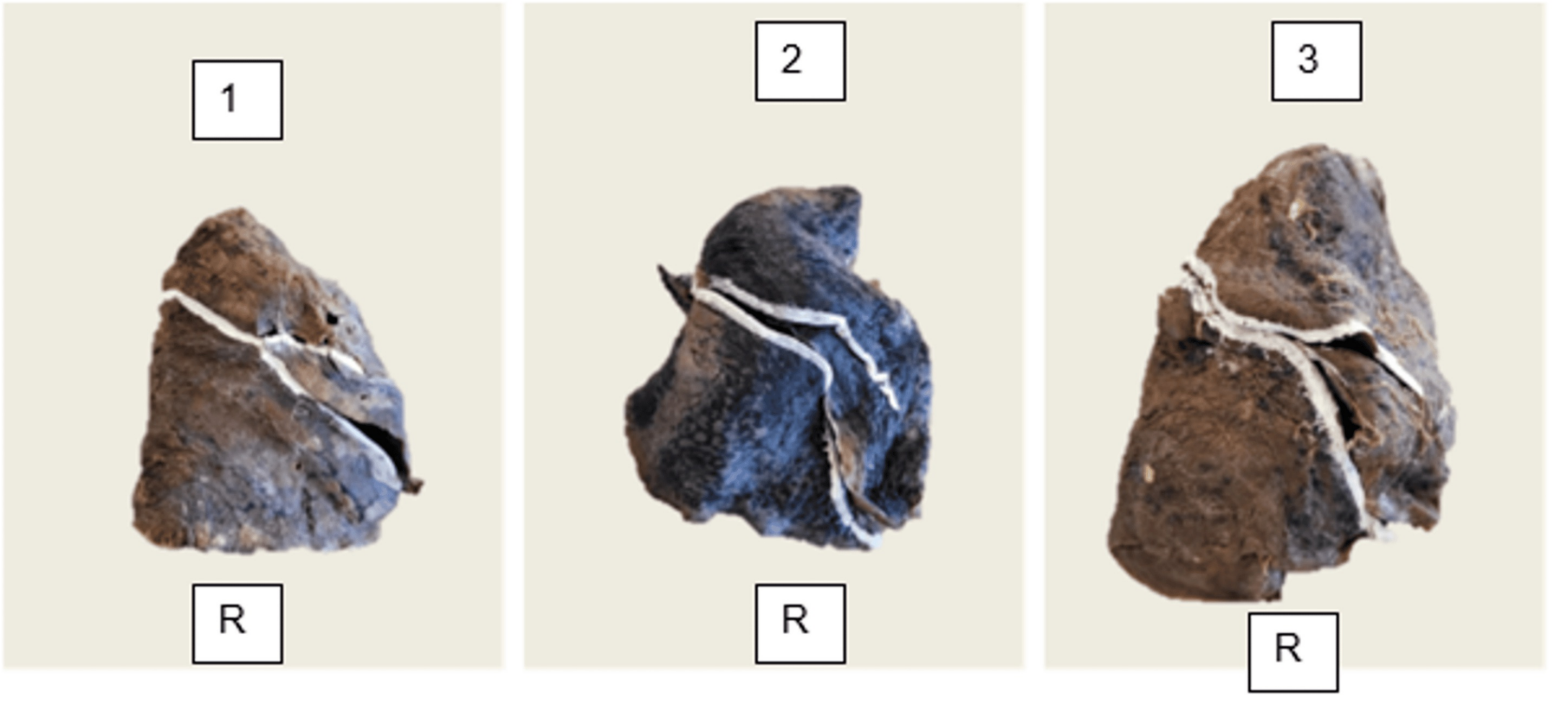
Right lung (R) with complete oblique fissure and incomplete horizontal fissure (n = 3; sub-panels numbered 1-3). The horizontal fissure fuses into the parenchyma after a partial course (Craig-Walker Grade I oblique, Grade III horizontal).

Finally, three lungs (10.0%) demonstrated a complete oblique fissure with an incomplete horizontal fissure, where the horizontal fissure disappeared within the parenchyma (Figure 4).

Summary of right lung findings

A classical pattern with complete oblique and horizontal fissures was observed in 19 lungs (63.3%), a bilobed configuration with a single complete oblique fissure in six lungs (20.0%), a complete oblique fissure with a fused horizontal fissure in two lungs (6.7%), and a complete oblique fissure with an incomplete horizontal fissure in three lungs (10.0%) (Table 1).

**Table 1:** Patterns of major fissures in right lungs (n = 30). * Fissure grade per Craig-Walker classification. † Rows marked with a dagger (n = 6 and n = 2) share the same Craig-Walker grade (Oblique I, Horizontal IV) and are pooled in all subsequent grade-based analyses (combined n = 8, 26.7%); they are listed separately here because they differ on gross inspection - complete absence of fissure versus shallow surface groove only.

Pattern of fissures (right lung)	Number (n)	Percentage (%)	Fissure grade*
Normal: complete oblique + complete horizontal	19	63.3	Oblique I, Horizontal I
Single complete oblique fissure (horizontal absent)	6^†^	20.0	Oblique I, Horizontal IV
Complete oblique + completely fused horizontal fissure (surface groove only)	2^†^	6.7	Oblique I, Horizontal IV
Complete oblique + incomplete horizontal fissure (fused after some distance)	3	10.0	Oblique I, Horizontal III

Patterns of major fissures in the left lungs

**Figure 5:**
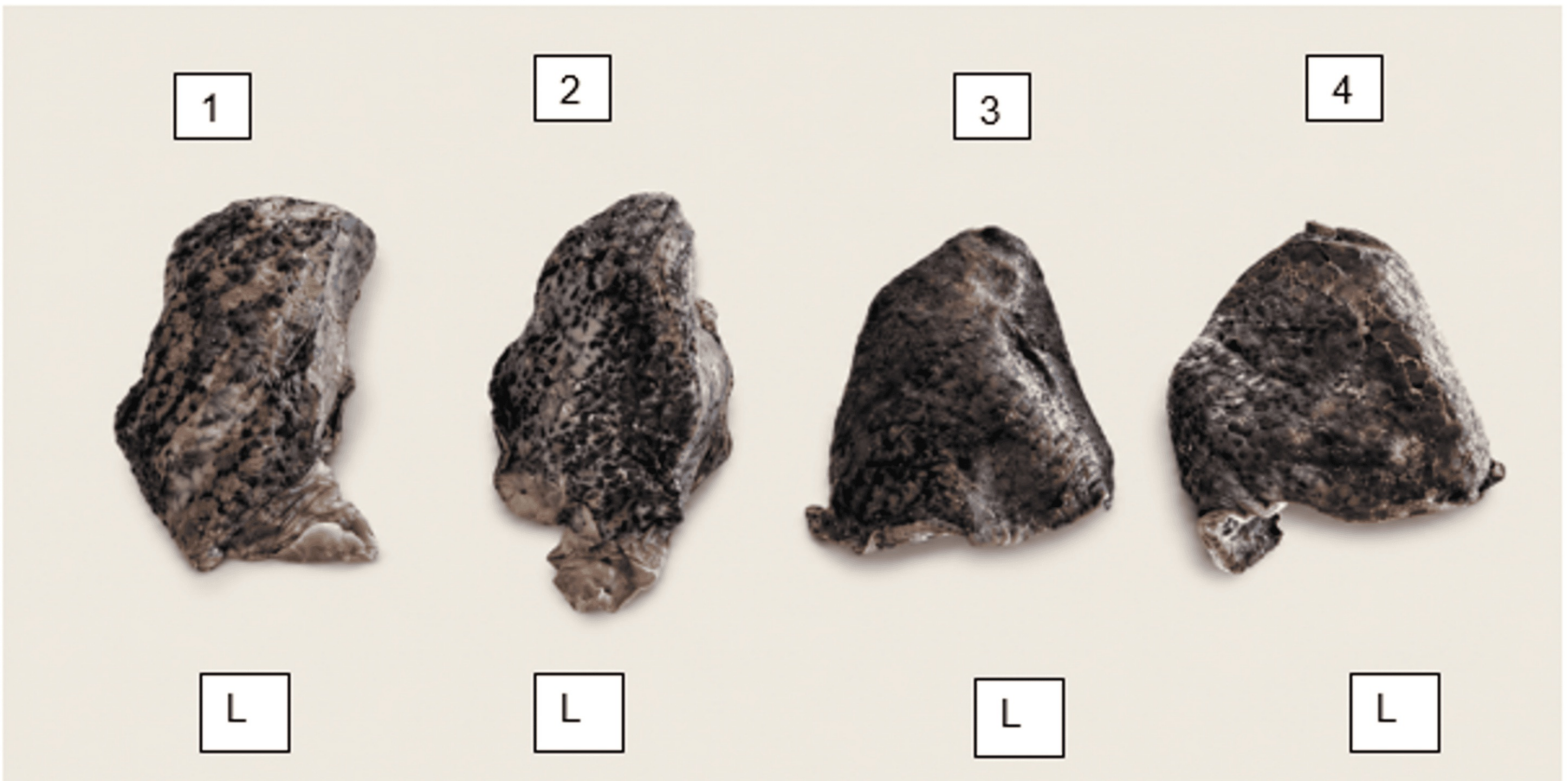
Left lung (L) specimens showing complete absence of the oblique fissure (n = 4; Specimens #33, #39, #47, #53 - corresponding to sub-panels 1-4, respectively). Upper and lower lobes are completely fused, forming a single undivided lung mass (Craig-Walker Grade IV oblique fissure).

Among the 30 left lungs examined, 25 lungs (83.3%) demonstrated a normal single complete oblique fissure. Conversely, four left lungs (13.3%) exhibited complete absence of any fissure, resulting in a single undivided lung mass (Figure 5). In accordance with the Craig-Walker system, these completely fused lungs were operationally classified as Grade IV oblique fissures, representing complete parenchymal fusion along the expected fissure plane.

**Figure 6:**
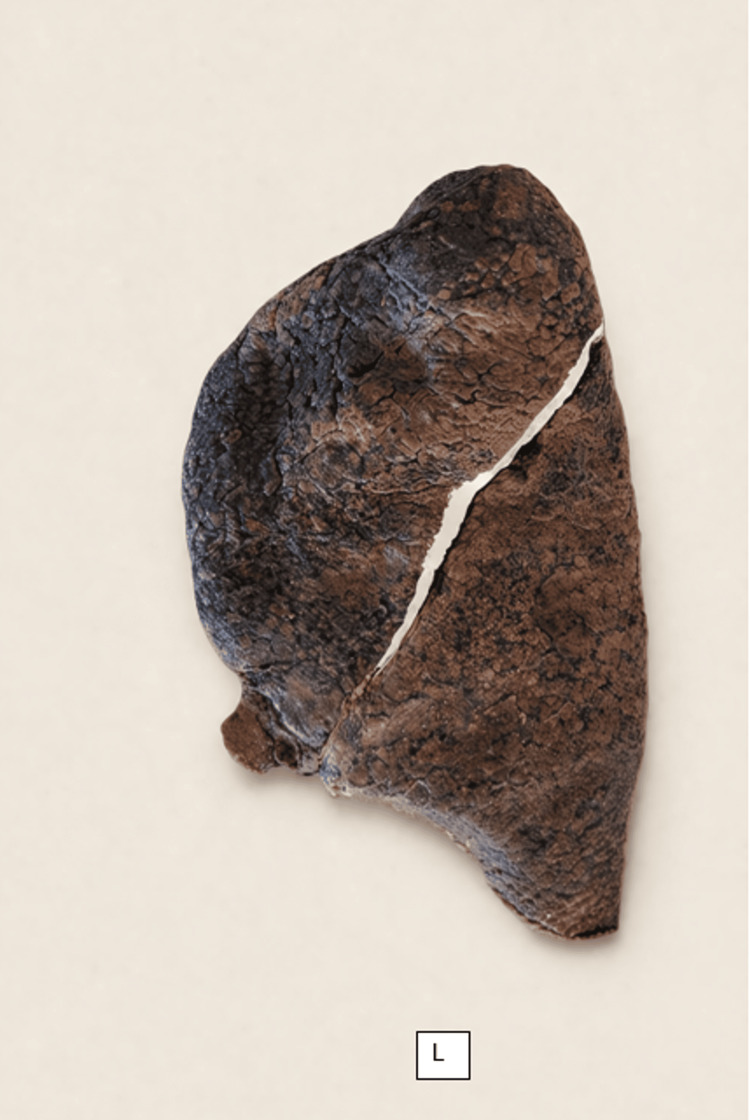
Left lung (L) Specimen #43 showing incomplete oblique fissure (Craig-Walker Grade III). The fissure is patent only in the middle portion and fused at the superior (upper lobe) and inferior (lower lobe) segments.

Additionally, one lung (3.3%) displayed an incompletely fused oblique fissure, fused at the superior and inferior segments but patent in the middle portion, corresponding to Grade III of Craig and Walker's classification (Figure 6).

No obvious accessory fissures (such as azygos, superior or inferior accessory fissures, or lingular fissures) were observed in this series. Similarly, no aberrant horizontal-type fissures were identified in the left lungs in this sample.

Summary of the left lung findings

A normal single complete oblique fissure (Oblique Grade I) was observed in 25 lungs (83.3%), absence of the fissure resulting in a single undivided lung mass (Oblique Grade IV) in four lungs (13.3%), and an incompletely fused oblique fissure (Oblique Grade III) in one lung (3.3%) (Table 2).

**Table 2:** Patterns of major fissures in left lungs (n = 30).

Pattern of fissures (left lung)	Number (n)	Percentage (%)	Fissure Grade
Normal: complete oblique fissure	25	83.3	Oblique I
No fissure (completely fused lung)	4	13.3	Oblique IV
Incomplete/fused oblique fissure (patent only in the middle)	1	3.3	Oblique III

Comparative analysis of the right and left lung patterns

The right lung exhibited greater variability in fissure completeness compared to the left lung, reflecting its more complex three-lobed structure. The predominance of classical fissure patterns in the right lung (63.3%) contrasts with the left lung, where 83.3% presented with the normal single complete oblique fissure. The proportion of lungs with normal fissure patterns was lower in right lungs compared to left lungs (63.3% vs. 83.3%), though the difference did not reach statistical significance (p = 0.1432, Fisher's exact test).

Considering all major fissures, complete oblique fissures (Grade I) were present in 55/60 lungs (91.7%), whereas the oblique fissure was Grade III in 1/60 (1.7%) and Grade IV in 4/60 (6.7%), all on the left side. For horizontal fissures, completeness was much lower: Grade I in 19/30 right lungs (63.3%), Grade III in 3/30 (10.0%), and Grade IV (absent) in 8/30 (26.7%) (Table 3).

**Table 3:** Fissure completeness using the Craig-Walker grading.

Fissure	Side(s)	Grade I, n (%)	Grade III, n (%)	Grade IV, n (%)	p-value
Oblique fissure	Right	30 (100.0)	0 (0)	0 (0)	0.0522 (Fisher's exact)
	Left	25 (83.3)	1 (3.3)	4 (13.3)	
Horizontal fissure	Right	19 (63.3)	3 (10.0)	8 (26.7)	Not applicable (right only)

Although Grade II fissures (complete cleft with parenchymal fusion at the base) form an important intermediate category in the Craig and Walker system, they were not encountered in the present series for either oblique or horizontal fissures. In this sample, fissural configurations were most often either fully complete (Grade I) or showed more advanced fusion (Grades III-IV); however, given the modest number of specimens, this pattern should be interpreted cautiously and may reflect sampling variation. These patterns are summarised by the Craig-Walker grade in Table 3.

Considering all major fissures together, complete oblique fissures (Grade I) were present in all 30 right lungs (100.0%) and in 25 of 30 left lungs (83.3%; p = 0.052, Fisher’s exact test); the remaining left lungs comprised one Grade III (3.3%) and four Grade IV (13.3%) oblique fissures, while no Grade III or Grade IV oblique fissures were observed on the right. This side-to-side difference did not reach statistical significance. Because of the limited sample size, these side‑to‑side comparisons have low statistical power, and non‑significant p values should not be interpreted as evidence of equivalence between sides. For horizontal fissures (right lungs only), Grade I completeness occurred in 19 of 30 lungs (63.3%), with Grade III in 3 of 30 (10.0%) and Grade IV (absent or fused) in 8 of 30 (26.7%).

## Discussion

In this cadaveric series, approximately one-third of right lungs (36.7%) and one-sixth of left lungs (16.7%) deviated from the expected Grade I fissure pattern, with complete oblique fissures present in 100% of right lungs but only 83.3% of left lungs. The higher proportion of abnormal fissure configurations in the right lungs did not reach statistical significance (p = 0.143), but the magnitude of the difference is consistent with a real anatomical contribution to the greater lobar complexity of the right lung. These findings extend prior anatomical work by providing side-stratified Craig-Walker grades and by documenting both bilobed right lungs and completely fused left lungs within a single contemporary Indian sample.

Our right-lung abnormality rate (36.7%) is broadly comparable to previously reported ranges. Murlimanju et al. observed incomplete or variant fissures in a similar proportion of right lungs in a cadaveric series [9], while Seleka et al. reported morphological variation in a comparable range in a South African sample [4]. The bilobed right-lung configuration observed in 20% of our specimens - complete oblique fissure with total absence of the horizontal fissure - matches the morphology most commonly implicated in intraoperative difficulties during right upper or middle lobectomy, in which the absence of an interlobar plane forces the surgeon to dissect through fused parenchyma [2,9,10]. The predominance of the classical single-oblique configuration in our left lungs (83.3%) is consistent with embryological expectation [1,5]; however, the 13.3% prevalence of a completely fused left lung is higher than in several prior series and deserves specific attention in pre-operative imaging for left-sided thoracic procedures.

Mechanistically, our findings align with the view that lobar fusion reflects delayed or incomplete obliteration of the intersegmental mesenchyme during the pseudoglandular stage of lung development [5,6]. The clustering of our specimens at either Grade I or Grades III-IV, with no Grade II, may indicate that partial basal fusion - the defining feature of Grade II - is a transient developmental intermediate rarely retained into adult life; alternatively, and given the limited sample size, this pattern may simply represent sampling variation, and larger cohorts are required to distinguish between these possibilities.

Incomplete fissures have important physiological as well as surgical consequences. Shared vascular and bronchial supply across a fused interlobar plane can alter regional ventilation-perfusion matching and may contribute to non-uniform collateral ventilation, with direct relevance to endobronchial valve therapy and bronchoscopic lung volume reduction, where an intact interlobar fissure is a prerequisite for successful atelectasis induction [3,11]. Quantitative computed tomography has emerged as a useful adjunct to cadaveric and intraoperative assessment, enabling preoperative estimation of fissure integrity and identification of patients at higher risk of prolonged air leak [11].

From a surgical standpoint, the importance of fissure completeness in video-assisted thoracoscopic surgery (VATS) and robotic lobectomy is well established. Patients with incomplete fissures (Craig-Walker Grade III-IV) have been reported to experience substantially higher rates of prolonged air leak, operative times that are appreciably longer, and higher rates of thoracotomy conversion compared with patients with Grade I anatomy [10,11]. The Grade III and Grade IV configurations we observed on the left side, together with the fused horizontal fissures seen in several right lungs, represent precisely the subset of cases in which fissure-first dissection fails and a tunnelling or stapler-based transparenchymal approach becomes necessary.

The Craig-Walker system thus functions not merely as a descriptive tool but as a bridge between cadaveric anatomy, cross-sectional imaging, and intraoperative decision-making. In our sample, 91.7% of all lungs had a Grade I oblique fissure - a proportion that reasonably predicts a technically favourable plane for the great majority of oblique-fissure-based procedures - while the Grade III and IV oblique fissures concentrated entirely on the left side (5/30, 16.7%) argue for routine pre-operative grading on CT for left-sided lobectomy candidates.

From a radiological perspective, the misinterpretation of fissure anatomy can lead to diagnostic pitfalls. Incomplete or accessory fissures may mimic pathological lines or lesions on imaging studies, potentially leading to unnecessary interventions or misdiagnoses [4]. The findings of our study underscore the importance of integrating anatomical knowledge with radiological interpretation to enhance diagnostic accuracy and improve patient outcomes.

The results are broadly consistent with previous cadaveric and imaging studies of pulmonary fissure anatomy. Murlimanju et al. reported complete oblique fissures in a proportion of cadaveric lungs very close to our overall Grade I rate [9]. The absence of any accessory fissure in our 60 lungs contrasts with the prevalence typically reported in Indian cadaveric series [5,8,9] and should be interpreted as a feature of this sample rather than a generalisable finding.

Limitations

This study has several important limitations. First, sex and age of the cadavers were not consistently recorded; because lung fissure morphology has been reported to vary with body size, ethnicity, and potentially sex [2-4], the absence of demographic data constitutes a meaningful constraint on interpretation and on comparison with demographically characterised series. Second, the cadavers were obtained through the routine body-donation and dissection programme of a single institution; the specific source, post-mortem interval, and duration of formalin fixation were not standardised or recorded at the level of individual specimens, which may introduce subtle differences in tissue texture and in the appearance of shallow fissures. Ethical approval and donor consent were obtained through the institutional body-donation pathway in accordance with the Anatomy Act, as stated in the ethics declaration. Third, the sample size (60 lungs; 30 per side) is modest, which limits statistical power for side-to-side comparisons and likely contributes to the absence of Grade II specimens in the series. Fourth, although the lungs were unpaired, right and left identity was confirmed at retrieval by hilar anatomy and by lobation rather than by fissure pattern alone, minimising - but not entirely excluding - the possibility of misclassification. Finally, although fissure grading was performed independently by two anatomists with discrepancies resolved by consensus, formal inter-observer reliability (kappa) statistics were not calculated. Larger, multicentric studies with complete demographic metadata and structured reliability assessment are needed to confirm and extend these observations.

Clinical implications

The findings have important clinical implications. Anatomical variations in fissure completeness can significantly influence surgical planning, particularly in procedures involving lobectomy or segmentectomy. Surgeons must be aware of these variations to anticipate potential complications and tailor their surgical techniques accordingly.

Additionally, the presence of anatomical anomalies, such as bilobed right lungs or undivided left lung masses, may affect pulmonary function and management of respiratory diseases. Clinicians should consider these variations when interpreting imaging studies and when diagnosing and treating lung-related conditions.

Despite these limitations, the study’s strengths include direct cadaveric examination, use of the Craig-Walker grading system for standardised fissure assessment, and detailed tabulation of clinically relevant fissural patterns.

## Conclusions

In summary, this descriptive cross-sectional study documents notable variability in the completeness of major pulmonary fissures between right and left cadaveric lungs, with a higher prevalence of deviations from the Grade I pattern on the right side and a small but clinically important proportion of completely fused left lungs. Recognition of graded fissure completeness using the Craig and Walker classification remains essential for surgeons and radiologists to optimise pre-operative planning and to minimise intraoperative complications in thoracic surgery.

## Appendices

**Table 4:** Cadaveric lung fissure study (n = 60).

Specimen #	Side	Pattern category	Oblique fissure grade	Horizontal fissure grade	Accessory fissures	Distinctive morphological features	Figure
1	Right	Normal classical (complete oblique + complete horizontal)	I	I	None	Well-preserved; sharp apex; smooth pleura	-
2	Right	Normal classical	I	I	None	Broad-based; mild fixation pallor; intact pleura	-
3	Right	Normal classical	I	I	None	Slightly elongated; pronounced diaphragmatic concavity	-
4	Right	Bilobed (single complete oblique; horizontal absent)	I	IV	None	Compact and rounded; horizontal fissure plane completely fused	Figure 2
5	Right	Normal classical - representative for Figure 1	I	I	None	Reference specimen; sharp apex, well-defined oblique and horizontal fissures	Figure 1
6	Right	Normal classical	I	I	None	Pleura mildly thickened over costal surface	-
7	Right	Normal classical	I	I	None	Robust upper lobe; clear cardiac impression	-
8	Right	Normal classical	I	I	None	Subtle interlobar adhesion at lateral end (not affecting grade)	-
9	Right	Complete oblique + completely fused horizontal (groove only)	I	IV	None	Shallow surface groove indicating intended horizontal plane; otherwise fused	Figure 3
10	Right	Normal classical	I	I	None	Slight rotation noted during photography due to specimen contour	-
11	Right	Bilobed	I	IV	None	Smooth uninterrupted parenchyma along expected horizontal plane	Figure 2
12	Right	Normal classical	I	I	None	Apex slightly rounded; horizontal fissure thin but complete	-
13	Right	Normal classical	I	I	None	Well-defined three-lobe pattern; minor pleural staining	-
14	Right	Complete oblique + incomplete horizontal (fused after some distance)	I	III	None	Horizontal fissure visible laterally then fades into parenchyma	Figure 4
15	Right	Normal classical	I	I	None	Preserved; mild dehydration of cut edges	-
16	Right	Normal classical	I	I	None	Apex sharply pointed; standard three-lobe pattern	-
17	Right	Bilobed	I	IV	None	Slightly broader than typical; horizontal fissure absent on all surfaces	Figure 2
18	Right	Normal classical	I	I	None	Pronounced cardiac notch on medial surface	-
19	Right	Normal classical	I	I	None	Fixation-related compression of upper lobe; grade unaffected	-
20	Right	Complete oblique + completely fused horizontal	I	IV	None	Shallow horizontal surface depression only; deep oblique fissure	Figure 3
21	Right	Normal classical	I	I	None	Standard morphology	-
22	Right	Bilobed	I	IV	None	Compact specimen; complete absence of horizontal fissure plane	Figure 2
23	Right	Normal classical	I	I	None	Elongated; well-defined fissures	-
24	Right	Complete oblique + incomplete horizontal	I	III	None	Horizontal fissure runs roughly two-thirds of expected course before fusing	Figure 4
25	Right	Normal classical	I	I	None	Small specimen; possibly female donor (sex not recorded)	-
26	Right	Normal classical	I	I	None	Standard adult morphology	-
27	Right	Bilobed	I	IV	None	Robust specimen; horizontal plane completely fused; deep oblique fissure	Figure 2
28	Right	Normal classical	I	I	None	Well-preserved; intact pleura throughout	-
29	Right	Complete oblique + incomplete horizontal	I	III	None	Horizontal fissure visible only in middle portion	Figure 4
30	Right	Bilobed	I	IV	None	Smaller-than-average specimen; horizontal fissure absent	Figure 2
31	Left	Normal (single complete oblique fissure)	I	NA	None	Well-defined cardiac notch; pronounced lingula	-
32	Left	Normal	I	NA	None	Standard two-lobe morphology	-
33	Left	Absent fissure (single undivided lung mass)	IV	NA	None	Parenchyma uninterrupted along expected oblique plane; no surface groove	Figure 5
34	Left	Normal	I	NA	None	Mild fixation-related distortion; oblique fissure clear	-
35	Left	Normal - representative for Figure 1	I	NA	None	Reference specimen; sharp apex; complete oblique fissure	Figure 1
36	Left	Normal	I	NA	None	Slightly compressed superior lobe; fissure morphology preserved	-
37	Left	Normal	I	NA	None	Pronounced cardiac impression	-
38	Left	Normal	I	NA	None	Standard adult morphology	-
39	Left	Absent fissure	IV	NA	None	Smooth uninterrupted parenchyma; no fissure line externally	Figure 5
40	Left	Normal	I	NA	None	Pleura mildly thickened in apical region	-
41	Left	Normal	I	NA	None	Elongated specimen; deep oblique fissure	-
42	Left	Normal	I	NA	None	Broad cardiac notch; well-formed lingula	-
43	Left	Incompletely fused oblique (patent only in middle)	III	NA	None	Fissure fused at superior and inferior segments; patent in middle 1/3 only	Figure 6
44	Left	Normal	I	NA	None	Standard morphology	-
45	Left	Normal	I	NA	None	Compact specimen; sharp apex	-
46	Left	Normal	I	NA	None	Slight rotation in photograph due to natural specimen contour	-
47	Left	Absent fissure	IV	NA	None	Completely fused; visible only as faint linear pleural mark	Figure 5
48	Left	Normal	I	NA	None	Mild dehydration of inferior border	-
49	Left	Normal	I	NA	None	Standard morphology; oblique fissure deep	-
50	Left	Normal	I	NA	None	Robust specimen; pronounced cardiac notch	-
51	Left	Normal	I	NA	None	Pleura intact; fissure clear	-
52	Left	Normal	I	NA	None	Smaller specimen; possibly female donor (sex not recorded)	-
53	Left	Absent fissure	IV	NA	None	Single undivided lung mass; no external fissure line; surface smooth	Figure 5
54	Left	Normal	I	NA	None	Standard morphology; pronounced lingula	-
55	Left	Normal	I	NA	None	Well-preserved; sharp apex	-
56	Left	Normal	I	NA	None	Mild pleural staining; fissure morphology preserved	-
57	Left	Normal	I	NA	None	Standard adult morphology	-
58	Left	Normal	I	NA	None	Slightly elongated specimen	-
59	Left	Normal	I	NA	None	Standard morphology; deep oblique fissure	-
60	Left	Normal	I	NA	None	Compact specimen; well-defined cardiac notch and lingula	-
